# Suicide rates in China, 2004–2014: comparing data from two sample-based mortality surveillance systems

**DOI:** 10.1186/s12889-018-5161-y

**Published:** 2018-02-13

**Authors:** Feng Sha, Qingsong Chang, Yik Wa Law, Qi Hong, Paul S. F. Yip

**Affiliations:** 1 Department of Social Work and Social Administration, The University of Hong Kong, Hong Kong, China; 2 Hong Kong Jockey Club Centre for Suicide Research and Prevention, The University of Hong Kong, Hong Kong, China; 3 Department of Public Courses, Shenzhen Institute of Information Technology, Shenzhen, China

**Keywords:** Epidemiology, Mental health, Ministry of Health, Center for disease control and prevention, Suicide, Surveillance

## Abstract

**Background:**

The decreasing suicide rate in China has been regarded as a major contributor to the decline of global suicide rate in the past decade. However, previous estimations on China’s suicide rates might not be accurate, since often they were based on the data from the Ministry of Health’s Vital Registration (“MOH-VR”) System, which is biased towards the better-off population. This study aims to compare suicide data extracted from the MOH-VR System with a more representative mortality surveillance system, namely the Center for Disease Control and Prevention’s Disease Surveillance Points (“CDC-DSP”) System, and update China’s national and subnational suicide rates in the period of 2004–2014.

**Methods:**

The CDC-DSP data are obtained from the National Cause-of-Death Surveillance Dataset (2004–2014) and the MOH-VR data are from the Chinese Health Statistics Yearbooks (2005–2012) and the China Health and Family Planning Statistics Yearbooks (2013–2015). First, a negative binomial regression model was used to test the associations between the source of data (CDC-DSP/MOH-VR) and suicide rates in 2004–2014. Joinpoint regression analyses and Kitagawa’s decomposition method are then applied to analyze the trends of the crude suicide rates.

**Results:**

Both systems indicated China’s suicide rates decreased over the study period. However, before the two systems merged in 2013, the CDC-DSP System reported significantly higher national suicide rates (IRR = 1.18, 95% Confidence Interval [CI]: 1.13–1.24) and rural suicide rates (IRR = 1.29, 95% CI: 1.21–1.38) than the MOH-VR System. The CDC-DSP System also showed significant reversing points in 2011 (95% CI: 2006–2012) and 2006 (95% CI: 2006–2008) on the rural and urban suicide trends. Moreover, the suicide rates in the east and central urban regions were reversed in 2011 and 2008.

**Conclusions:**

The biased MOH-VR System underestimated China’s national and rural suicide rates. Although not widely appreciated in the field of suicide research, the CDC-DSP System provides more accurate estimations on China’s suicide rates and is recommended for future studies to monitor the reversing trends of suicide rates in China’s more developed areas.

## Background

Suicide is a major global health issue. A report on suicide prevention by the World Health Organization (“WHO”) estimated a total number of 804,000 suicide deaths worldwide in 2012, which had fallen by about 9% since 2000 [1]. The WHO Mental Health Action Plan 2013 aims to achieve a further 10% reduction in suicide rate by 2020 as the global goal [2]. The decline of global suicide rate since 2000 was mainly driven by a drop of 47% in the suicide rate in low and middle-income countries in the Western Pacific Region, where China accounted for more than 80% of its population [1]. Considering China’s huge population and overwhelming contribution to the reduction of the global suicide rate, precise appraisals on China’s suicide rates are of great importance to the accuracy of global suicide rate estimations, and also, whether China maintains a declining suicide rate, is crucial in achieving the global reduction goal.

The WHO report estimated that the suicide rate in China has declined by 59.6%, from a standardized age suicide rate of 19.4 per 100,000 population in 2000 to 7.8 per 100,000 population in 2012 [1]. However, the Global Burden of Disease (“GBD”) study reported a drop of 42.6% from 17.0 to 9.7 in this same period [3]. One of the reasons for the discrepancies between these two studies is that their China’s suicide data are from two different sample-based mortality surveillance systems. The WHO report based mainly on the Ministry of Health’s Vital Registration (“MOH-VR”) System [4]; whereas the GBD study was from the Chinese Center for Disease Control and Prevention’s Disease Surveillance Points (“CDC-DSP”) System [5]. Since the mortality data from the MOH-VR System were first reported to the WHO in 1987, information on suicides had already been updated by many studies [6–16]. The MOH-VR System did not have a random sampling design and moreover, oversampled the better-off population in the east China cities and their peripheral areas with relatively good reporting systems. Therefore, the MOH-VR System was not representative of the poorer rural and urban areas [8, 17, 18]. An earlier study, 15 years ago, suggested that the effect of the lack of representativeness of the MOH-VR System was not great when compared with the representative CDC-DSP System [8]. However, both these two systems have changed significantly since then (details to be elaborated in the data part) and there is new evidence that areas with lower socio-economic circumstances (“SEC”) are more likely to have higher suicide rates within both the rural and urban areas in China [19]. Hence, studies using the data from the MOH-VR System may underestimate China’s general suicide rate compared to the CDC-DSP System. In this paper, suicide rates reported by the MOH-VR System are compared to those from the CDC-DSP System and their differences examined.

Both the GBD study and the WHO report found a significant decline of suicide rate in China, from its climax in the 1990s and 2000s [1, 20], yet, less attention has been given to its recent national and subnational suicide trends. The great drop in China’s suicide rate was mainly associated with rapid urbanization and a substantial reduction in the number of people having convenient access to lethal pesticides and the improvement of employment opportunities [21]. However, as the rural-to-urban ratio of suicide rates reduces over time [19], the marginal benefit of urbanization on suicide rate has simultaneously been diminishing. In the more developed areas like the east coast, further urbanization and rapid change in society may even induce stress and adjustment problems which are not conducive to the promotion of well-being [16]. Moreover, if the elderly suicide rate still remains much higher than other age groups as is present [22], China’s fast-ageing population would inevitably have a negative impact and contribute to the increase of suicide rates [16]. Therefore, the diminishing benefit of urbanization and the increasing negative impact of ageing could reverse the decreasing national suicide trend in the future, which may have already taken place in some of the most developed and ageing areas, such as the east and central urban areas of China.

This study aims to suggest that the CDC-DSP System provides more accurate information on China’s suicide rates between 2004 and 2014 and presents that national and subnational suicide trends in the period under study using the CDC-DSP System. The hypotheses of this study are that (i) nationally and within both rural and urban areas, the MOH-VR System reports lower suicide rates than the CDC-DSP System; and (ii) according to the CDC-DSP System, there are some reversing suicide trends in the more developed urban areas, such as the east and central urban areas in China.

## Methods

### Sources of data

In this study, suicide counts from 2004 to 2014 classified by sex, quinquennial age-group, residence (rural/urban), and geographic location (east/central/west) population size with corresponding population size from the CDC-DSP System reported in the National Cause-of-Death Surveillance Dataset (2004–2014) are used [23, 24]. The CDC-DSP System was established as both a national and regional (i.e. the east, central and west parts in both rural and urban areas) representative mortality surveillance system commencing 1990, covering approximately 1% of the total population and expanded to 161 surveillance points nationwide in 2004 (covering a total population of 6%) [22].

The sex, age and residence (rural/urban) specific suicide rates from the MOH-VR System in 2004–2014 were drawn from the Chinese Health Statistics Yearbooks (2005–2012) [25] and the China Health and Family Planning Statistics Yearbooks (2013–2015) [26]. Established in the1950s, the MOH-VR System covered 36 cities and 90 counties from 15 out of the 31 provinces and municipalities, the total samples of which accounted to roughly 8% of the total population of Mainland China by 2000 [18]. By 2012, the system has expanded to include 319 surveillance points (total population coverage of 17%), mostly in the east and central areas [18]. In 2013, the CDC-DSP and MOH-VR Systems were merged into the Integrated National Mortality Surveillance (“INMS”) System [18]. Thus, data from the two sources should be the same after 2013.

### Statistical methods

Poisson Regression Modelling was first used to test the association between the source of data (CDC-DSP/MOH-VR) and suicide rates with controlling variables such as year, sex, quinquennial age-group and residence (rural/urban), because Poisson regression assumes a Poisson distribution that tends to fit suicide rate data better than the linear regression model. However, as the unconditional mean of suicide rates was much lower than its variance, indicating that suicide rates were over-dispersed, a Negative Binomial Regression Model is used instead. Discrepancies of the two systems were quantified by the Incident Rate Ratio (“IRR”) and its 95% Confidence Interval (95% CI). Interactions between the sources of data and all other variables were also tested. Poisson Regression and Negative Binomial Regression analyses were performed using PROC GENMOD in statistical software SAS, version 9.4 for Windows.

Further, Joinpoint Regression is used to analyze the trends of the sources of data (CDC-DSP/MOH-VR) on the residence (rural/urban) and geographic locations (east/central/west) of the crude suicide rates. Based on Segmented Linear Regressions with the log of suicide rates as the dependent variable and the years as independent variable, the models were used to estimate the number and locations of join points and the annual percent change in rates between the points. Joinpoint Regression analysis was conducted using Joinpoint software version 4.0.4 by the Nation Cancer Institute, US [27]. Kitagawa’s Decomposition Method [28] was then used to quantify the contributions of age composition and sex and age specific suicide rates to the upward trends of the crude suicide rates.

## Results

### Comparing suicide rates from the CDC-DSP and MOH-VR systems

**Fig. 1:**
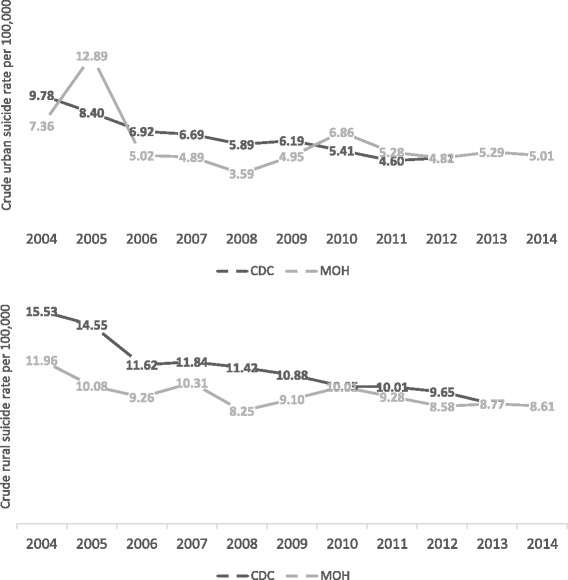
Rural and urban suicide rates from the CDC-DSP and MOH-VR Systems, 2004–2014. Data of crude rural and urban suicide rates per 100,000 population from the CDC-DSP and MOH-VR Systems were presented

Figure 1 showed the variations of rural and urban suicide rates from the two systems between 2004 and 2014. In addition to the total suicide rates in both the rural and urban areas, specific rates of sex, age, and residence (rural/urban) were also the same in those two systems in 2013 and 2014 as the two systems were merged into one in 2013 [18]. Before 2012 however, the MOH-VR System consistently reported less rural suicides than the CDC-DSP System. Moreover, fluctuations of the urban suicide rates around 2005 and 2010 and the rural suicide rates around 2007 and 2010 in the MOH-VR System did not exist in the CDC-DSP System. The results of the Negative Binomial Regression (Table 1) showed that with the same controlling time, age group and sex, the CDC-DSP System in general reports significantly higher suicide rates than the MOH-VR System (IRR = 1.18, 95% CI: 1.13–1.24). The interaction between residence and data source was also significant, which indicates that the CDC-DSP System reports a significantly higher rural suicide rate than the MOH-VR System (IRR = 1.29, 95% CI: 1.21–1.38). No statistical differences, however, were found on interactions between the data source and other variables.

**Table 1:** Incident Rate Ratio (“IRR”) of suicide rates

Variables	IRR (95% CI)	*P*
Years*	0.93 (0.92, 0.93)	<.0001
Age groups*	1.29 (1.28, 1.30)	<.0001
Sex (ref = male)		<.0001
Female	0.79 (0.75, 0.83)	
Residence (ref = urban)		<.0001
Rural	1.93 (1.84, 2.03)	
Source (ref = MOH-VR)		0.04
CDC-DSP	1.18 (1.13, 1.24)	
Residence*Source		0.0003
Urban CDC-DSP (ref = Urban MOH-VR)	1.08 (1.00, 1.16)	
Rural CDC-DSP (ref = Rural MOH-VR)	1.29 (1.21, 1.38)	

Year, age, sex and residence specific suicide rates from the MOH-VR and CDC-DSP Systems are compared in Table 1. Years and age groups are continuous variables. Years range from “1 = 2004” to “9 = 2012” and age groups range from “1 = 10–15 years” to “16 = 85+ years”

### Secular trends in suicide rates: Joinpoint regression analyses

**Fig. 2:**
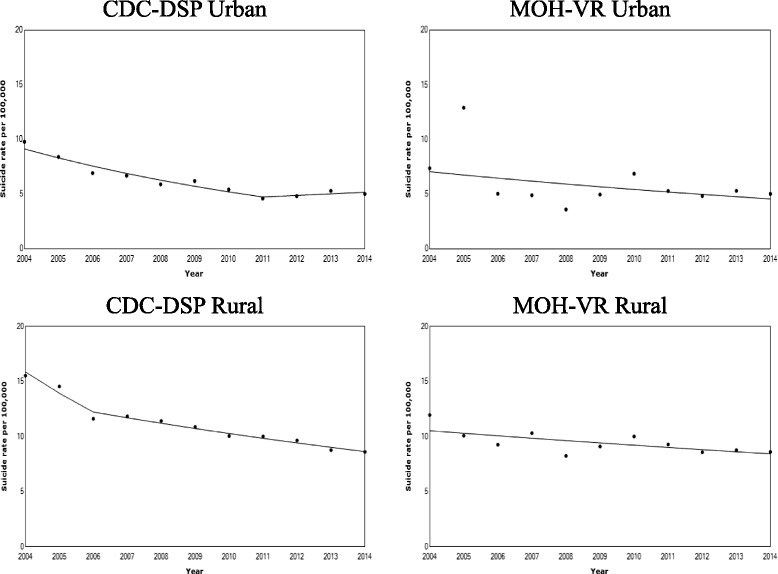
Rural and urban suicide rates from the CDC-DSP and MOH-VR Systems, 2004–2014. Data of crude rural and urban suicide rates per 100,000 population with line segments of the joinpoint regression models from the CDC-DSP and MOH-VR Systems were presented

Figure 2 and Table 2 summarized the results of the Joinpoint Regression analyses of the crude urban and rural suicide rates reported from the CDC-DSP and MOH-VR Systems in the period 2004–2014, showing estimated years when the suicide trend changed (Join Points /“JP”s) and the Annual Percent Change (“APC”) in suicide rates between those join points. The crude suicide rates from the CDC-DSP System indicated one significant joinpoint in 2011 (95% CI: 2006–2012) and 2006 (95% CI: 2006–2008) on the urban and rural suicide rates respectively (Table 3), while the data of the MOH-VR System showed no significant join point but a steadily declining trend.

**Table 2:** The results of Joinpoint Regression analyses on secular trends of urban and rural suicide rates

	Segment 1		Segment 2
	APC (95% CI)	JP (95% CI)	APC (95% CI)
Urban			
CDC-DSP	−9.0 (− 11.8, − 6.0)	2011 (2006, 2012)	3.0 (−8.7, 16.2)
MOH-VR	−4.3 (− 10.5, 2.4)		
Rural			
CDC-DSP	−12.2 (− 18.0, −6.1)	2006 (2006, 2008)	−4.3 (− 5.0, −3.5)
MOH-VR	− 2.2 (−3.9, − 0.5)		

Summary of the Annual Percent Change (“APC”) in Crude Suicide Rates Joinpoints (“JP”s) on the trends in the urban and rural suicide rates and their 95% Confidence Intervals (“CI”s) using the data from the CDC-DSP and MOH-VR Systems (2004–2014). APC coefficients are the annual percent changes in suicide rates in the years between the specific joint points. Negative coefficient indicates downward trends; positive coefficient indicates upward trends. JPs are years when changes in suicide trends occur

**Table 3:** The results of joinpoint regression analyses on secular trends of six regions

	Segment 1		Segment 2
	APC (95% CI)	JP (95% CI)	APC (95% CI)
East			
Urban	− 8.3 (− 10.9, 5.6)	2011 (2009, 2011)	3.8 (− 5.4, 13.9)
Rural	− 5.9 (− 6.8, 5.1)		
Central			
Urban	− 16.3 (− 25.9, − 5.4)	2008 (2006, 2012)	0.5 (− 5.1, 6.6)
Rural	−5.5 (− 6.5, − 4.5)		
West			
Urban	−7.8 (− 9.0, − 6.6)		
Rural	−18.1 (− 32.3, − 0.9)	2006 (2006,2012)	− 2.3 (− 4.2, − 0.3)

Summary of the Annual Percent Change^1^ (“APC”) in suicide rates Joinpoints^2^ (“JP”s) on the trends in Crude Suicide Rates and their 95% Confidence Intervals (“CI”s) for the population age 10+ in the three regions of China using the data from the CDC-DSP System (2004–2014). APC coefficients are the annual percent changes in suicide rates in the years between the specific joint points. JPs are years when changes in suicide trends occur

**Fig. 3:**
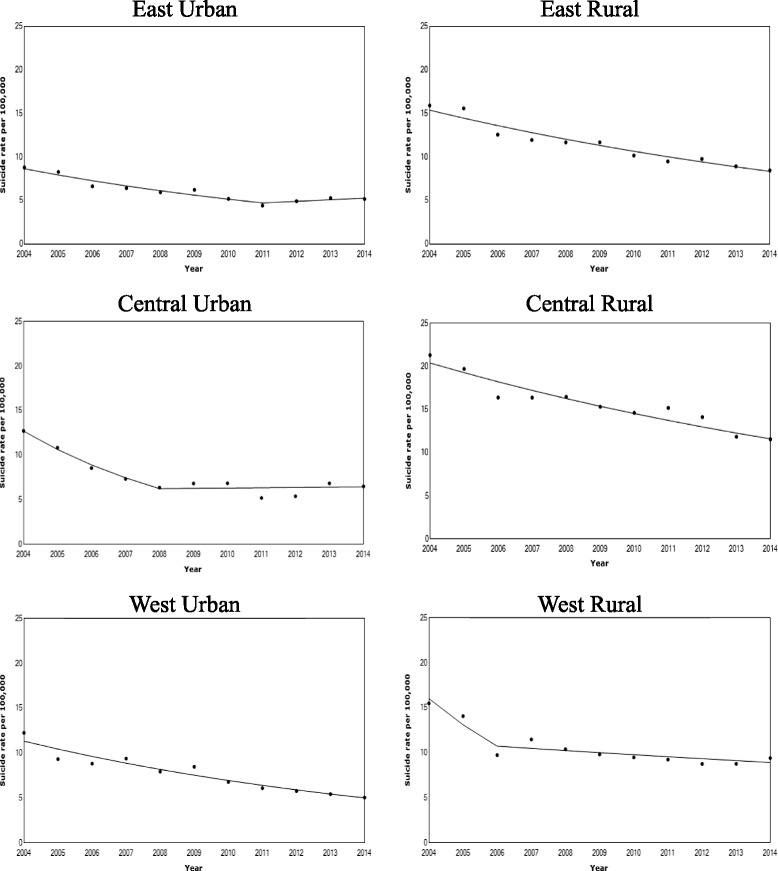
Rural and urban suicide rates in six regions from the CDC-DSP System, 2004–2014. Data of crude rural and urban suicide rates per 100,000 population aged 10+ with line segments of the joinpoint regression models from the CDC-DSP System were presented

Figure 3 and Table 3 showed the results of joinpoint regression upon further exploring the subnational crude suicide rates (age = 10+) trends from the CDC-DSP System. From this, in 2011, reversing trends in suicide rates on the east urban areas (95% CI: 2009–2011), and in the central urban areas in 2008 (95% CI: 2006–2012) were identified.

### Decomposition of the reversing suicide rate trends

Table 4 showed the relative contributions of changes in population proportion and age-specific suicide rates to the reversing suicide rate trends in the east urban and central urban areas in 2011–2014 and 2008–2014, respectively. During 2011–2014, the crude suicide rate (aged 10+) in the east urban area increased by 17.2%. The increasing middle-aged (aged 35–64) male suicide rate (10.4%) and the growing proportion of the elderly population (7.5% + 6.1% = 13.6%) are the main driving factors of the increase in the suicide rate. During 2008–2014, the crude suicide rate (aged 10+) in the central urban areas increased by 2.1%. Although the decline of suicide rates among the middle-aged male (− 2.2%) and female (− 4.5%) would have reduced the suicide rate by 6.7%, yet the increasing elderly suicide rate among the male (3.3%) and the growing proportion of the middle-aged and elderly population (2.3% + 1.2% + 0.6% + 0.9% = 5.0%) drove the total suicide rate up by 8.3%.

**Table 4:** Decomposition of the reversing suicide rate trends in the east and central urban regions

	Male		Female	
	Due to change in population proportion	Due to change in suicide rates	Due to change in population proportion	Due to change in suicide rates
East Urban (2011–2014)				
10–34	0.6%	− 1.7%	0.3%	− 1.1%
35–64	−1.8%	10.4%	−0.9%	3.0%
65+	7.5%	−2.8%	6.1%	− 2.5%
Central Urban (2008–2014)				
10–34	−0.2%	0.2%	−0.1%	− 0.1%
35–64	2.3%	−2.2%	1.2%	−4.5%
65+	0.6%	3.3%	0.9%	0.7%

Percentage of relative contributions are presented

## Discussions

Our findings suggest that the CDC-DSP System reports an average of 18% higher suicide rates across all the sex-, age- and residence-specific groups than the MOH-VR System in the period from 2004 to 2012, while in the same period, the CDC-DSP System also reports 29% higher in rural suicides than the MOH-VR System. After 2013 however, the data from the two systems are identical. Quality of the data is also assessed by examining their trends from 2004 to 2014 [29]. The reported trend of urban suicide rates from the MOH-VR System fluctuates vastly around 2005 and 2010, yet in the CDC-DSP System, they are relatively stable. Moreover, the turning points in 2011 (95% CI: 2006–2012) for urban areas and 2006 (95% CI: 2006–2008) for rural areas from the CDC-DSP System do not appear in the MOH-VR System.

The inconsistencies between the two systems are probably due to the lack of representativeness and in the definitions of urban and rural areas in the MOH-VR System before 2012, when its coverage was biased towards the better-off population of the east China cities and their peripheral rural areas. According to the results of this study (Fig. 3) and previous studies using the CDC-DSP data [19, 22, 30], suicide rates in the east were always lower than the average of the central and west areas. Therefore, the MOH-VR System systematically underestimated the total suicide rate in the period from 2004 to 2012. Moreover, comparing with the CDC-DSP System, the definitions of urban and rural regions in the data from the MOH-VR System are not consistent. In 2005, the definition of urban and rural regions in the MOH-VR changed from crude administrative classification of cities (including districts and counties administrated by cities) and counties to a more detailed demarcation of districts and counties [26]. Therefore, the counties administrated by cities which were used to be classified as urban areas before 2005, were since 2005, regarded as rural areas, which may explain for the huge fluctuations of urban suicide rates from the MOH-VR System at that time. The CDC-DSP System however, has consistently adopted the more sophisticated definitions of urban and rural since 2004. Therefore, the CDC-DSP System is considered to possess a more representative sampling method, with a more consistent definition on urban and rural, thus furnishes more reliable suicide data than the MOH-VR System.

Since 2011, the upward urban suicide trend reported by the CDC-DSP System is also noteworthy. The rural suicide rates in China used to be two- to three-fold greater than the urban rates, a huge discrepancy between the rural and urban suicide rates, while in most of the western counties, rural and urban suicide rates are almost the same [21]. Many scholars opine the great drop in China’s suicide rate in the past decade attributable to urbanization and urban migration brought about by the rapid social and economic developments [8, 9, 12, 13, 31]. However, the protective effect of urbanization on suicides has been recently plateauing [16], which is also quite in tune with the findings of reversing urban suicide rates in this study. In China, the east region is most developed, followed by the central region and then the west. The upward suicide trend in the east urban region since 2011 is mainly due to the increasing suicide rates among the middle-aged male and the increasing population proportion of the elderly. The loss of young population and increasing suicide rates among the elderly male contribute to the increase of the suicide rates in the central urban region since 2008. The results are also in tune with previous discussions on the possibility of the reversing trends of suicide rates [16]. The first wave of baby boomers born in the 1950s and 1960s have started to reach the age of 60 in the 2010s, thus, China is facing an accelerating ageing population [22]. Around 2010, its impact on suicide rates has already surfaced in the relatively developed urban areas like the east and central urban regions of China. Moreover, the massive migration of the central urban young population to the east coast left many older adults behind, resulting in the central urban areas suffering not only from ageing population but also increasing suicide rates among the elderly male since 2008. It is thus foreseen that there will be a further increase in national suicide rates alongside the ageing population pattern in the rural region. In addition, economic crisis is also a widespread concern that may increase suicide rates, as economic downturns always go hand in hand with high unemployment rate, which associates with the increase in suicides [32–34]. Although since China’s economic retardation and transformation in 2012 the official urban registered unemployment rate in China still remains low (4.1%), however, the real unemployment rate may be escalating as the people who have lost their jobs lack the incentive to register because of the low levels of unemployment benefits, and the social stigma of being unemployed [35]. Like many other developed Asian regions such as Hong Kong, South Korea and Japan in the 1997–1998 economic crisis, the rise of suicide rates among the middle-aged male in the east areas may be associated with the economic downturn, job uncertainty and the ensuing increasing unemployment rate.

Nonetheless, the CDC-DSP System is not without its own limitations. Same as many other mortality surveillance systems, its information regarding suicides also suffers from the issues of under-reporting and misclassifications. The latest evaluation on the CDC-DSP System estimated an overall under-reporting rate of 12.9% in the period from 2009 to 2011, and the under-reporting rate was higher in the west (18.8%) than the east (10.1%) and central (11.2%) regions [36]. However, because of sex, quinquennial age-group, residence (rural/urban), and geographic location (east/central/west) specifics, under-reporting rates are not available on general mortality, hence, the under-reporting rate cannot be taken into account in this study. Therefore, the lower suicide rate in the west regions may be partially attributable to its higher under-reporting rate. The most recent evaluation of misclassification of suicides within the CSC-DSP System dates back to the period from 1995 to 2000, which stated that 1.9% suicides should be redistributed to other external causes, with 5.4% ‘other’ external causes, 48.6% ‘unknown’ external causes, and 15.0% deaths resulted from psychiatric disorders be re-distributed to suicides [37]. However, there is no updated evaluation on the misclassification of the expanded CDC-DSP System since 2004. Therefore, an assumption cannot be made that the misclassification patterns would be the same, as the evaluation was conducted almost two decades ago. In this study, focus is placed more on the differences of the raw data and their trends between the two systems namely, the CDC-DSP and MOH-VR Systems, assuming the misclassification and under-reporting rates remain stable during the period of 2004–2014.

## Conclusions

In conclusion, the results of the comparison between the CDC-DSP and MOH-VR Systems indicate that previous studies using the data from the MOH-VR System may systematically underestimate China’s suicide rates, especially in the rural region. The statements in the WHO 2012 report on suicides that neither of those two datasets was systematically higher in quality than the other is doubtful [4]. After the two systems merged in 2013, the new INMS System will be the best source for reporting China’s suicide rates, yet, the data from the CDC-DSP System before 2013 are recommended to be compared with those from the new INMS System to monitor the changes in future studies. The main reason for the merits of the CDC-DSP System being less appreciated in the field of suicide research hinges on the simple fact that it is not as easily accessible as the MOH-VR System. Data from the CDC-DSP System are currently only attainable in a series of published yearbooks in Chinese [23], which makes it extremely difficult for scholars to access. Nonetheless, the CDC-DSP System not only provides unbiased and consistent estimations of China’s suicide rates, but is also representative of subnational information, which shows that China’s suicide rates in the more developed areas (east and central urban regions) had ceased dropping and started to rise around 2010. China’s suicide rates are already so low that the increasing trends of subnational suicide rates could be an early sign of reversing its national rate of suicides, and therefore warrants continuous monitoring by future studies.

## Abbreviations

APC: Annual percent change
CDC-DSP: Center for disease control and prevention’s disease surveillance points
CI: Confidence intervals
GBD: Global burden of disease
IRR: Incident rate ratio
JP: Join point
MOH-VR: Ministry of Health’s Vital Registration
SEC: Socio-economic circumstances
WHO: World Health Organization
